# Experiences that matter in bipolar disorder: a qualitative study using the capability, comfort and calm framework

**DOI:** 10.1186/s40345-023-00293-9

**Published:** 2023-04-20

**Authors:** J. E. Siegel-Ramsay, S. J. Sharp, C. J. Ulack, K. S. Chiang, T. Lanza di Scalea, S. O’Hara, K. Carberry, S. M. Strakowski, J. Suarez, E. Teisberg, S. Wallace, J. R. C. Almeida

**Affiliations:** 1 Department of Psychiatry and Behavioral Sciences, Dell Medical School, The University of Texas at Austin, Austin, TX USA; 2 Value Institute for Health and Care, Dell Medical School, The University of Texas at Austin, Austin, TX USA; 3 The City University of New York School of Labor and Urban Studies, New York, NY USA; 4 Dell Medical School and McCombs School of Business at the University of Texas, Austin, TX USA

**Keywords:** Bipolar disorder, Psychosocial, Treatment, Qualitative, CCC framework, Capability Comfort and Calm Framework

## Abstract

**Background:**

When assessing the value of an intervention in bipolar disorder, researchers and clinicians often focus on metrics that quantify improvements to core diagnostic symptoms (e.g., mania). Providers often overlook or misunderstand the impact of treatment on life quality and function. We wanted to better characterize the shared experiences and obstacles of bipolar disorder within the United States from the patient’s perspective.

**Methods:**

We recruited 24 individuals diagnosed with bipolar disorder and six caretakers supporting someone with the condition. Participants were involved in treatment or support services for bipolar disorder in central Texas. As part of this qualitative study, participants discussed their everyday successes and obstacles related to living with bipolar disorder during personalized, open-ended interviews. Audio files were transcribed, and Nvivo software processed an initial thematic analysis. We then categorized themes into bipolar disorder-related obstacles that limit the patient’s capability (i.e., function), comfort (i.e., relief from suffering) and calm (i.e., life disruption) (Liu et al., FebClin Orthop 475:315–317, 2017; Teisberg et al., MayAcad Med 95:682–685, 2020). We then discuss themes and suggest practical strategies that might improve the value of care for patients and their families.

**Results:**

Issues regarding capability included the struggle to maintain identity, disruptions to meaningful employment, relationship loss and the unpredictable nature of bipolar disorder. Comfort related themes included the personal perception of diagnosis, social stigma and medication issues. Calm themes included managing dismissive doctors, finding the right psychotherapist and navigating financial burdens.

**Conclusions:**

Qualitative data from patients with bipolar disorder helps identify gaps in care or practical limitations to treatment. When we listen to these individuals, it is clear that treatments must also address the unmet psychosocial impacts of the condition to improve patient care, capability and calm.

## Background

Over one year, it is estimated that 2–3% of adults in the United States have bipolar disorder. (Kessler et al. 2005; Merikangas et al. 2007). The condition impairs life functioning and quality (e.g., reduced social support, increased risk of suicide) (Bensing 2000; Michalak et al. 2008; Gitlin and Miklowitz 2017), with a majority (83%) of individuals with bipolar disorder reporting severe functional impairment. (Kessler et al. 2005) Treatment for the condition also comes at an increased cost. At The University of Texas, individuals with bipolar disorder had an almost two-fold increase in medical insurance costs compared to controls (Bipolar = $52,000 vs. Control = $27,000). Individuals with a history of hospitalization for bipolar disorder incurred medical costs similar to life-threatening medical conditions such as cancer or myocardial infarction (Bipolar = $108,000; Cancer = $105,000; Myocardial Infarction = $103,000) (Leung et al. 2021) Given the condition’s prevalence, severity, and cost, it is imperative that treatment dollars be targeted toward interventions that improve symptom severity, life quality and function for the patient.

In many research and clinical settings, the value of an intervention for bipolar disorder is quantified by the reduction in manic or depressive symptoms, but broader metrics of the condition are essential to understanding the full value of a treatment (Chatterton et al. 2017; Malhi et al. 2017, 2018). Wallace and Teisberg designed “The Capability, Comfort and Calm framework” as an alternative to symptom-based care measurements. This framework focuses on the clinical and life priorities of people with a shared set of persistent, long-term or palliative care needs. Capability outcomes relate to the ability to function and maintain identity by doing things important to the individual. Comfort outcomes reduce the physical, mental, and emotional suffering caused by a medical condition. Calm refers to outcomes that relate to the experience of receiving health care. (Wallace and Teisberg 2016, 2017; Liu et al. 2017; Teisberg et al. 2020).

Health care centered around assessing and understanding patient needs and goals improves patient experience and outcomes (Wallace and Teisberg 2016, 2017; Teisberg et al. 2020; Guzik et al. 2021). Patient surveys can monitor experience, but qualitative research offers a distinct personalized perspective that compliments quantitative methods. Open-ended and conversational research is essential to focus research and clinical care toward overlooked or misunderstood outcome priorities for patients and their families and further encourage positive patient-clinician relationships. For example, patient interviews in inpatient psychiatric settings in the United Kingdom called attention to the patient-focused treatment priority of living outside the care facility. These findings led to updated treatment plans and encouraged care that focuses on long-term patient priorities. (Wallang et al. 2018; Andrews et al. 2019).

Evidence suggests that the treatment for individuals with bipolar disorder is not fully addressing their long-term needs (Chatterton et al. 2017; Warwick et al. 2019). Individuals with bipolar disorder report severe disruptions to life, psychosocial dysfunction, social stigma and loss of purpose or identity (Pollack and Aponte 2001; Sajatovic et al. 2005; Inder et al. 2008; Jönsson et al. 2008; Venkataraman and Ackerson 2008; Delmas et al. 2011; Ward 2011; Crowe et al. 2012; Borg et al. 2013; Hawke et al. 2013; Madden et al. 2020). Treatment-related distress is also reported in association with inadequate provider knowledge, limited treatment options and reduced patient involvement or trust (Fisher et al. 2018; Maassen et al. 2018; Zolfi Kashani et al. 2020).

There are currently only a handful of qualitative research studies in individuals with bipolar disorder living in the United States (e.g., Pollack and Aponte 2001; Sajatovic et al. 2005; Venkataraman and Ackerson 2008; Ward 2011; Nestsiarovich et al. 2017). Many of these studies focused on specific subgroups of bipolar disorder, including individuals with comorbid substance abuse disorders (Ward 2011), mothers (Venkataraman and Ackerson 2008), rapid-cyclers (Sajatovic et al. 2005) or inpatients (Pollack and Aponte 2001). Studies reported the need for individualized, holistic patient-centered support (Pollack and Aponte 2001; Sajatovic et al. 2005; Venkataraman and Ackerson 2008). The experience of bipolar disorder was also associated with negative stigma, disruption to identity and reduced socio-occupational life quality (Sajatovic et al. 2005; Ward 2011). Nestsiarovich and colleagues conducted focus groups in Montana, New Mexico and California and identified medication decisions and access to quality mental health care as overarching core difficulties for individuals with bipolar disorder and their families during their treatment journey (Nestsiarovich et al. 2017) A more recent qualitative phone study of individuals with bipolar disorder also reported severe financial burdens and insurance complications that resulted in inadequate psychotherapy treatment and increased stress leading to more severe symptoms.(Madden et al. 2020). These study findings suggest universal and American-specific experiences of bipolar disorder.

The Bipolar Disorder Center at Dell Medical School prioritizes value-based health that is outcome-driven, evidence-based and patient-centered. Understanding patient experience is central to delivering patient-based care (Wallace and Teisberg 2016, 2017; Liu et al. 2017; Teisberg et al. 2020; Almeida et al. 2022). In 2019, the Bipolar Disorder Center partnered with researchers at the Value Institute for Health and Care, a joint enterprise between The University of Texas at Austin’s Dell Medical School and McCombs School of Business, to conduct a qualitative study in people diagnosed with bipolar disorder. We were interested in understanding lived experiences of individuals with bipolar disorder and their families in Texas, which ranks last among other U.S. states for mental health access.(Reinert et al. 2021) As a first step toward improving patient-valued care, we conducted this qualitative study to identify gaps in treatment and focus treatment on outcomes that matter most to this unique population. Our goal was to characterize unique and clinically overlooked aspects of bipolar disorder care that may be typical of many individuals receiving outpatient treatment for the condition within the United States.

## Methods

### Study participants

We recruited 24 adults over 18 years of age (female n = 16) diagnosed with bipolar disorder from the Bipolar Disorder Center at Dell Medical School and other bipolar disorder support services within central Texas. Recruited individuals with bipolar disorder were between 18 and 58 years of age. Parents of individuals with bipolar disorder were also recruited at these locations to gain some perspective on a caregiver’s experience of the condition. Each person who participated received a grocery store gift card. The Office of Research Support and Compliance Institutional Review Board at The University of Texas at Austin approved this study.

### Study design and data collection

Two doctorate-level researchers with training in qualitative research (JS and CU) designed and conducted individual interviews in English. According to grounded theory methodology (Glaser and Strauss 2017), interview questions were designed without prior hypotheses, and the goal was to understand patient experience through observation. Questions were broad, open-ended and variable between individuals and groups. Some examples of questions posed by interviewers include:

1. “What does a typical day look like for you?
2. “What are the main challenges you face in your experience with bipolar disorder?”
3. “What types of support work for you?”
4. “What does success look like for you in your journey with bipolar disorder?”
5. “What is the role of the various health care professionals that you see for bipolar disorder?
6. “What is missing in your journey experience living with bipolar disorder?”

Research interviewers (JS, CU) facilitated five in-person groups, each consisting of 3–5 people. Group sessions were stratified by age (18–34 and 35–58). Group interviews lasted between 90–120 min. Researchers conducted in-person individual interviews with those who were unable or uncomfortable meeting in a larger group setting (n = 12). Individual interviews lasted 60–90 min, but otherwise, discussion guides for individual interviews followed the same principles as the group sessions: semi-structured with open-ended questioning to allow the participant to select experiences and stories essential and relevant to their diagnosis of bipolar disorder. Interviews were conducted in a non-clinical conference room at Dell Medical School or a local public library. Data from 29 participants were audio-recorded and transcribed verbatim. One participant session was manually recorded through note-taking. The two interviewers (JS, CU) debriefed participants after each interview for 30–60 min and discussed significant themes and topics presented during the session. Researchers took notes during both the sessions and the debriefs.

### Analysis

After the audio files were transcribed, researchers (JS, CU) conducted an initial thematic analysis of the data collected from individuals with bipolar disorder using NVivo, a computer-assisted qualitative data analysis software tool (Dhakal 2022). Researchers included all verbal interview texts from individuals with bipolar disorder in the thematic analysis. Following the initial coding, researchers categorized themes raised by participants within the *Capability, Comfort*, and *Calm* framework (Wallace and Teisberg 2016, 2017; Liu et al. 2017; Teisberg et al. 2020). Researchers recruited a small number of parents (20% of the total cohort) and therefore did not include these interviews in the NVivo analysis. Results from parental interviews were considered in supplement to the individual patient interviews and presented when they informed or illuminated the patients' insights.

## Results

### Capability—ability to support identity through work and relationships

#### Struggle to maintain an identity

“…When I said [bipolar is] confusing, what I meant is it’s confusing in that I really don’t know who I am. I question every conversation in every social environment that I’ve been in. Did I say too much? Was I talking too fast? Was I really me? … I don’t really get to just be. It’s always judging.”—patient.

For individuals with bipolar disorder, variable and changing thoughts and emotions combined with a negative perception by their community weakened their sense of identity. Participants often had difficulty differentiating thoughts and behaviors that were attributed to bipolar disorder from neurotypical emotions and cognitions. For example, some participants described pursuing a relationship or career goals during a manic episode but having those goals dissipate with the resolution of the manic episode. Another participant shared a story where she surprised her daughter with cake and balloons. Her daughter immediately questioned if this was a symptom of mania. Some participants discussed how they enjoyed mania and that treatment made them feel “boring.” Several participants shared the experience of people assuming they were “crazy” or dangerous based solely on the diagnosis of bipolar disorder.

#### Meaningful employment

“I've literally been fired from every job that was fire-able."—patient.

“[My goal is] to build a career in what I want. Not just random meaningless jobs forever. Something that I’m committed to. It’s pretty important.”—patient.

Almost all participants described disruptions to school and/or work. Many participants discussed the desire for financial independence and a personally meaningful job. Occupational disruptions not only happened during mood episodes but also during periods of euthymia. Participants attributed employment difficulties to medication side effects on energy and cognition and the interference of monthly physician or weekly psychotherapy appointments. Some participants described having to take employment below what they were qualified or capable of performing. However, one participant used his experience with bipolar disorder to become a peer support specialist, a position where people with lived experience offer support and counseling to others with that shared life experience.

#### Loss of relationships

"… I lost everything. I lost my family. It was my nuclear family, my mom, that reported me to Childhood Protective Services (CPS). I had lost that support system as well. I lost my husband, I lost my kids. Then with the divorce, I lost his family. I didn't have any friends. I just hit rock bottom."—patient.

“…having a wrap around that treats the whole family…things that benefit the whole family system to make sure that you’re all recovering together”—caregiver.

Many participants had relationship difficulties, often attributed to behavior during mood episodes. Participants spoke of difficulty maintaining social networks of friends, romantic partners, and family. One participant perceived his symptoms would always be a barrier to maintaining social networks, and another participant shared his fear that he would be unable to start a family. Participants who had intact support emphasized the importance of family support to their well-being.

#### Unpredictable nature of bipolar disorder

"…out of the blue, all of a sudden after 15 years of really great stability, everything just changed really. I don't know why."—patient

Participants struggled with the unpredictability of bipolar disorder and routinely talked about "stability." Stability to some participants meant emotional stability, which leads to stable behavior, work and relationships. Other participants expressed that stability would be feeling "normal" and not thinking about stability. Most participants believed stability arises from medication and psychotherapy, but more than one participant refused medication as they felt it worsened their quality of life.

### Comfort—reducing physical and mental suffering

#### Perception of diagnosis as indeterminate and unscientific

"Well something else is that it's not cancer. Or this is part of why it's hard to accept a bipolar diagnosis because there's not a blood test. There's not a physical marker for 'this is what it means to have bipolar disorder’. You look at the symptoms, and then that determines whether or not you have it."—patient

Many participants found the nature of psychiatric diagnosis and the inconsistency of diagnosis between providers distressing and disconcerting. Multiple providers evaluated many participants and received different diagnoses and treatment recommendations. Participants discussed currently having comorbid diagnoses of anxiety, ADHD, and PTSD. Some expressed frustration that providers did not clearly communicate the diagnostic process or criteria.

#### Social stigma—lack of public awareness and education

"It's like, "Hey, I have bipolar." And they want to get away from you. I'm not dangerous, but there's just the negative stigma attached to it."—patient.

“When our daughter was diagnosed with these mental health issues, we didn’t get casseroles, but if she had been diagnosed with cancer, we would have. That whole stigma that goes with it. So there’s shame.”—caregiver.

Most participants experienced stigma in social and professional settings (e.g., impaired ability to obtain jobs, employers concerned about work disruptions). Some participants wished for more public awareness about bipolar disorder to reduce the stigma. Several individuals reported that family members refused to accept a bipolar disorder diagnosis of their loved one since they were not "crazy" or violent.

#### The trials of medication

"These meds are making me sick. It's making this, and this, and this, and this happen. …And so, I just stopped taking the medication because I thought I was going to die."—patient.

Many participants discussed antidepressant medication, typically prescribed prior to a bipolar diagnosis, worsening depression, or inducing mania. Participants experienced trialing multiple medications due to ineffectiveness and often accepted side effects as preferable to mood episodes. Common side effects cited were impaired cognition, blunted emotions, and avolition. Some participants expressed that finding the proper medication regimen could take months. The potential side effects and unpredictability of medication treatment had physical and emotional consequences, leaving many participants feeling an overall lack of support.

### Calm—healing and consistent providers

#### Dismissive doctors

"I was confused about the diagnosis…how that related to me, how they [providers] could just give me medicine without offering any path to recovery. And then angry that they would do that."—patient.

Participants often felt that providers dismissed concerns about diagnosis and treatment. Some participants wanted a more robust explanation and support at the initial diagnosis. Many participants noted that short appointment times limited the ability to address all concerns. Several individuals reported limited choices or loss of providers due to insurance restrictions or a general lack of psychiatrists in the community. Lack of support and consistency made it difficult for many participants to find a long-term provider they could trust, a challenging proposition for a condition in which the care offered by providers is highly variable.

#### Finding the right psychotherapist

"They're like, 'Yep, you're bipolar. Here's medicines and a list of counseling services you can call.' It's like six pages long, and all heavily lists. And so, that was incredibly frustrating to call all of those numbers and get voicemails, and leave messages, and then weeks and months later get calls back."—patient.

Many participants found finding an available psychotherapist complex and confusing, even though most were interested in psychotherapy and believed it would benefit their well-being. They reported limited choices due to insurance and inadequate guidance from providers. Securing regular care felt like an insurmountable obstacle for those underinsured and/or lacking the time and resources necessary to find a therapist.

#### Financial burden

"I don't think I can get my medications or therapy unless I am gainfully employed. I think I'm just going to be basically working to pay for my medications and my medical health treatment. That's why I think there's no hope for me in this country."—patient.

“He knows that he’s always going to have to have some kind of health insurance forever. I”ll have him covered until he’s 26, but that’s about it.”—caregiver.

Participants shared positive and negative experiences with services funded by local and federal agencies providing care to vulnerable populations, including individuals without insurance, poor-quality insurance, or financial hardships. The high-cost burden of treatment often restricted access to psychiatrists, psychotherapists, or medications. Some participants reported that they could not afford tolerable and effective medication due to a lack of insurance or high out-of-pocket cost. Participants also shared difficulty navigating the disability system, which would help access care, address psychosocial stressors, and avoid homelessness.

## Discussion

This study aimed to better understand the priorities and unmet needs of individuals diagnosed with bipolar disorder. When we listen to these individuals, it is clear that greater focus needs to be placed on the unmet psychosocial impacts of the condition. Participants reported that bipolar disorder negatively impacts their *capability* by limiting meaningful social and work-related functions, impacting their identities and creating instability. The primary difficulties regarding *comfort* included inconsistent and obscure clinical diagnostic processes, trials identifying adequate psychotropic treatment and social stigma of the condition. Finally, *calm* related themes included a lack of support and time from their clinicians, difficulties identifying and obtaining psychotherapy and financial difficulties related to treatment. Although clinical symptoms likely impact these patient priorities and need to be managed, it is clear that to improve the outcomes that matter most to individuals living with bipolar disorder, therapeutic management needs to be attentive and responsive to restoring function, removing treatment obstacles and developing trusted provided relationships. We present an overview of the themes identified within this research and suggested interventions that might improve the patient experience in Table 1.

**Table 1:** The CCC framework with interventions

Treatment obstacles identified	Potential interventions
Capability	
Struggle to maintain identity	- Support social and professional functioning - Psychotherapy to challenge feelings of inadequacy
Meaningful employment	- Cognitive remediation - Potential pharmacology targeting cognitive impairments - Address residual depressive symptoms and side effects of medication - Career support services
Loss of relationships	- Family-focused therapy - Caregiver support - Social skills training - Interventions that address comorbidities - Earlier intervention
Unpredictable nature of disorder	- Access to care, medicine and psychotherapy services to improve symptom control
Comfort	
Perception of diagnosis as indeterminate and unscientific	- Providers must acknowledge patient’s experience of misdiagnosis and heterogenous nature of bipolar disorder - More research focusing on biomarkers and risk factors for bipolar disorder - Robust psychoeducation - More time with providers
Social stigma—lack of public awareness and education	- Increase public education - Collaborative patient-clinician discussions to develop trust and acknowledge patient experiences
The trials of medication	- Evidence-based treatment - Clearer medication guidelines and mental health research for prescribers - Increased public education - Realistic and trusting patient-clinician collaborations about medication decisions
Calm	
Dismissive doctors	- Increased time and better communication with providers - Holistic support from social workers and mental health support teams to cover limitations in clinician availability
Finding the right psychotherapist	- Improved support to navigate psychotherapy in clinic - Institutional changes to mental health care in the United States to reduce obstacles to treatment - Train more psychologists to cover shortages
Financial burden	- Early intervention prior to more severe outcomes and treatment costs - Employers and providers provide social service and insurance support - Increased local and federal support - Consider disparities in education, geography and income when providing financial support and assistance

### Improving capability by supporting and maintaining employment, personal identity, symptom stability and social support

In agreement with other qualitative studies (Jönsson et al. 2008; Michalak et al. 2008; Crowe et al. 2012; Maassen et al. 2018), participants in this study experienced employment difficulties and a desire for meaningful employment. One participant reported employment success in a role in which the experience of bipolar was considered a strength. Practical and individual-specific career support services from both employers and mental health providers can make work more obtainable, help maintain personal identity and lessen the financial stressors of the condition. (Drake et al. 2013; Filia et al. 2021) Compared to other medical settings, people accessing mental health services are more likely to view these services as a pathway to resources outside the health care system, such as housing, education, and employment (Easter et al. 2016). Providers and treatment teams should be aware that people may have this expectation for help. Case managers, peer support specialists and social workers could also provide employment support (e.g., navigating human resources or providing interview training).

In agreement with other studies (Marvel and Paradiso 2004; Bora et al. 2013; Siegel-Ramsay et al. 2022), participants also reported cognitive impairments, which may further exacerbate employment difficulties. Cognitive impairment may be associated with medication side effects, residual depressive symptoms or neurocognitive decline (Marwaha et al. 2013; Kim et al. 2018; Lu et al. 2022). Cognitive remediation, behavioral interventions to improve cognition, has been extensively studied and validated for schizophrenia. Recently, there has been increased interest in cognitive remediation for bipolar disorder (Vieta et al. 2013; Kim et al. 2018), although more research is required to establish its effectiveness. Other interventions include more aggressive treatment of depressive symptoms or pharmacotherapy targeting cognitive impairments (Gitlin and Miklowitz 2017).

Individuals with bipolar disorder also reported difficulty with their identity and an overall lack of stability. In another qualitative study in bipolar disorder, individuals reported that dysregulated and contradicting emotional experiences combined with self-doubt in their abilities contributed to a reduced sense of self. Investigators also reported that individuals with bipolar disorder reported a stronger sense of self when they had greater control of their symptoms and learned to integrate the conflicting nature of their condition into their identity (Inder et al. 2008).

Participants in the study also identified social support as a deciding factor in their quality of life. People with bipolar disorder experience more significant disruptions in relationships, higher divorce rates and are less likely to get married (Kogan et al. 2004; Granek et al. 2016). Greater focus and characterization of the larger functional and psychosocial difficulties typical to the condition are an essential first step toward improving outcomes and quality of life for those with bipolar disorder.

Although not the primary focus of this study, it is also essential to remember that a diagnosis of bipolar disorder impacts not only the patient but also close family members and friends. The clinical realities of care and increased healthcare costs impact families of an individual with bipolar disorder (Chatterton et al. 2008; Delmas et al. 2011). Caregivers of individuals with bipolar disorder report high levels of distress concerning the patient's symptoms and long-term function. Reported distress increases when the diagnosed individuals have more significant functional deficits, increased symptoms, or cycling. Caregivers also report that their reduced mental and physical health when caring for someone with bipolar causes significant distress. (Reinares et al. 2006) Most critically, individuals with bipolar disorder have more negative outcomes when their caregiver reports a higher subjective burden of care. (Perlick et al. 2004; Reinares et al. 2006; Hadryś et al. 2011).

Regarding social and self-identity, psychotherapy for bipolar should focus on recognizing and managing symptoms and social functioning (Geddes and Miklowitz 2013) while also challenging the person's association of a psychiatric diagnosis with incompetency and inadequacy (Yanos et al. 2010). Social skills training has also shown some benefits in individuals with bipolar disorder (Aruldass et al. 2022). Additionally, treatments that address comorbidities that impact social relationships or identity (i.e., personality disorders) should be incorporated into the treatment of bipolar disorder for the individual (Bateman et al. 2015). Finally, researchers and clinicians should focus on identifying and treating the condition at earlier stages to lessen the progressive nature of the illness and more quickly moderate the symptoms that contribute to diagnosed individuals' social, personal identity and employment issues.(Correll et al. 2007; Berk et al. 2010; Tse et al. 2014; Yildiz 2021).

Treatment providers must also acknowledge that patients' support networks directly impact patient outcomes. For example, increased socio-occupational functional impairments contribute to increased suicide rates and worsen the clinical symptom severity in individuals with bipolar disorder. (Johnson et al. 2016; Dome et al. 2019) Interpersonal relationships and work may also make life more meaningful, contributing to positive psychosocial functioning and suicide resiliency (Kleiman and Beaver 2013; Dezutter et al. 2014). Given that individuals with bipolar disorder experience a 20- to 30-fold increase in suicide and 9–20 years reduced life expectancy compared to the general population (Chesney et al. 2014; Plans et al. 2019), treatments shown to improve psychosocial functioning might fundamentally save lives. Evidence suggests that family-focused therapy, an intervention now being offered at the Bipolar Disorder Center at Dell Medical School, reduces caregiver distress and outcomes for the patient (e.g., fewer relapses) by strengthening family dynamics (Miklowitz et al. 2003; Reinares et al. 2004; Miklowitz and Chung 2016). When appropriate to the patient, the treatment team should help both the person and their family with psychoeducation or provide psychotherapy for the bipolar disorder diagnosis.

### Improving comfort through a more precise characterization of the disorder, addressing stigma and medication consensus

People who receive a diagnosis of bipolar disorder may have mixed experiences during diagnosis, including relief, confusion or disappointment resulting from the increased burden and social stigma. The stigma of bipolar disorder is a common experience for patients with the illness and often comes with a loss of social support and occupational success, increased symptom burden and lower quality of life (Hawke et al. 2013; Warwick et al. 2019). Some participants also discussed some of the positive aspects of bipolar disorder (Venkataraman and Ackerson 2008; Russell and Moss 2013), which providers must acknowledge (e.g., increased creativity) (Galvez et al. 2011; Greenwood 2017) when discussing the adverse outcomes of mania (Gruber 2011).

Misdiagnosis is common for individuals with bipolar disorder. On average, a diagnosis occurs five to ten years after initial symptom onset (Hirschfeld et al. 2003; Baldessarini et al. 2007). Providers often overlook short-lived symptoms of mania until they severely impact the individual's life. (Judd et al. 2002, 2003) Additionally, symptoms overlap between bipolar disorder and other mental health conditions (e.g., major depressive disorder and borderline personality disorder) (American Psychiatric Association 2013). Misdiagnosis and symptom overlap create confusion and distrust in mental healthcare, but this is rarely acknowledged or addressed in treatment. Better understanding the patient’s experience of misdiagnosis could strengthen providers’ ability to build trust with patients. In the longer term, biomarkers and risk factors that might more clearly differentiate bipolar disorder from other conditions need continued development and direct clinical application to improve diagnosis and treatment (Rowland and Marwaha 2018; Siegel-Ramsay et al. 2021).

Medications for bipolar disorder can be intolerable or ineffective for some individuals. (Sajatovic et al. 2005; Jann 2014; Greene et al. 2018). Prescribing can vary significantly by provider preferences, and a systematic review showed that most patients (85%) are on two or more medications, while 36% are on four or more medications (Fornaro et al. 2016). Medication adherence may be as low as 25%, and one-third of people with bipolar disorder have somebody in their core social network discouraging medication use. A narrative review that included qualitative studies identified side effects, complex regimens, lack of insight, or poor relationships with prescribers as factors impacting adherence (Jawad et al. 2018).

A lack of consensus for the appropriate treatment combined with the misdiagnosis of bipolar disorder can contribute to difficulties in finding the correct medication regimen. Lithium should be the first-line treatment for bipolar disorder (Won and Kim 2017; Yatham et al. 2018), but providers are increasingly hesitant to prescribe it (Bohlken et al. 2020; Rhee et al. 2020). Psychopharmacology should be supported by evidence-based research addressing the emotions, side effects, and perceived effectiveness of treatment through collaborative decision-making that involves patients and promotes better adherence and satisfaction to medication treatment (Fisher et al. 2018). Increased mental health education and clear guidelines from bipolar disorder scientific societies are necessary to improve diagnosis, treatment and stigma among providers and the public. For example, the National Network of Depression Centers Task force has begun efforts to educate providers about lithium (lithium works for bipolar disorder: why aren’t we using it? 2023). Treatment decisions should also include the individual with bipolar disorder, and the clinician should ask about barriers to treatment and compliance. When bipolar disorder treatment includes patient education, trust and involvement, there is a significant increase in long-term outcomes and individual independence (Stafford and Colom 2013).

### Improving calm with more supportive provider relationships, coordinated care and decreased financial burden

Participants in our study confirmed the importance of solid provider relationships to their well-being and reported that communication and adequate time contributed to a more positive relationship with providers. They described an inability to see preferred providers and psychologists due to insurance, cost, availability or information barriers. Another U.S. based qualitative study by Madden and colleagues (2020) reported that insurance restrictions and financial burdens of care result in limiting access to appropriate providers or medications, decisions to underutilize clinical appointments and longer-term treatments like psychotherapy. These obstacles make adequate treatment more difficult and increase stress levels (Bjoerkman et al. 1995; Madden et al. 2020; Wharam et al. 2020; Leung et al. 2021). Within the United States, practitioner shortages and insufficient medical insurance coverage contribute to inadequate mental health care (Bishop et al. 2014, 2016, p. 20; Xu et al. 2019). Higher unemployment rates and reduced social support typical of individuals with bipolar disorder may further exacerbate these U.S. specific financial and insurance-based stressors.

Mental health treatment within the U.S. has failed individuals requiring long-term mental health care. Even with new policies to support mental health care, private healthcare companies' financial support falls well below care for physical health coverage (Pelech and Hayford 2019; Whitney and Peterson 2019). Given these financial pressures, programs focusing on early intervention and adequate patient-focused care may reduce more severe and expensive outcomes such as hospitalization and inpatient support (Bessonova et al. 2020; Teisberg et al. 2020; Leung et al. 2021).

Providers, employers and treatment teams should also be sensitive to these ongoing financial pressures. Support and guidance with social service resources and insurance can have a direct impact on improving patient care and outcomes. Some participants in our study reported positive experiences in obtaining financial support through local or federal agencies, suggesting that continuing to improve these services can be critical to the most vulnerable individuals with bipolar disorder. Access to care is also impacted by education level, geography and income (Björkman et al. 1995; Karanti et al. 2021), and it is also critical that we consider these disparities between individuals (Kim et al. 2017).

### Limitations

This study aimed to better understand the overlooked treatment needs for individuals with bipolar disorder living in central Texas. After analyzing the data, we decided to publish our findings because we felt the themes identified from this research would better our understanding of unmet patient needs within the US. Unfortunately, the original data collection did not include detailed demographic, diagnostic and clinical information of participants. Limitations of our study also included a small sample size and recruitment of participants only receiving care. The experience between bipolar disorder subtypes or those not requiring ongoing clinical care may differ from those reported in this study. Participants lived in the same state but in different cities; each city may have unique resources and challenges. Recruitment methods may also be biased toward participants who want to share that they are overly satisfied or dissatisfied. Participants all spoke English as a primary language, limiting the ability to generalize data to patients speaking other languages. Although many of our findings are supported by other qualitative research studies in individuals with bipolar disorder, our findings should be replicated in larger, more heterogeneous samples of individuals with the condition.

## Conclusions

Care designed to help patients achieve the outcomes that matter most to them is integral to meeting patient needs and improving care value. Gathering insight by listening to the stories and experiences of people living with bipolar disorder can help guide future research, care and the use of resources for the condition. We suggest some strategies that might improve the value of care based on Capability, Comfort, and Calm themes discussed by individuals with bipolar disorder. Implementing change may be as simple as reviewing social history or adding questionnaires that assess psychosocial function. Capability, Comfort and Calm outcomes could be used as criteria for the design of care services, which would provide a holistic and unique approach compared to a more typical fractured and inconsistent care delivery for bipolar disorder in the United States. In the future, we intend to gather more detailed qualitative information to help design measures and implement interventions to continue delivering cost-effective care that most improves the health outcomes for people with bipolar disorder.

## Data Availability

All data is confidentially stored and de-identified data may be available for publisher’s review.
